# Metal(loid)s in *Cucurbita pepo* in a Uranium Mining Impacted Area in Northwestern New Mexico, USA

**DOI:** 10.3390/ijerph16142569

**Published:** 2019-07-18

**Authors:** Christine Samuel-Nakamura, Felicia S. Hodge, Sophie Sokolow, Abdul-Mehdi S. Ali, Wendie A. Robbins

**Affiliations:** 1 School of Nursing, University of California, Los Angeles (UCLA), 4-246 Factor Bldg., Mailcode 691821, Los Angeles, CA 90095, USA; 2 School of Nursing, University of California, Los Angeles (UCLA), 5-940 Factor Bldg., Mailcode 691921, Los Angeles, CA 90095, USA; 3 School of Nursing, University of California, Los Angeles (UCLA), 5-238 Factor Bldg., Mailcode 691921, Los Angeles, CA 90095, USA; 4 Department of Earth and Planetary Sciences, University of New Mexico, Northrop Hall MSCO 3-2040 Albuquerque, NM 87131, USA; 5 Center for Occupational and Environmental Health Fielding School of Public Health, Environmental Health Sciences, University of California, Los Angeles (UCLA), 5-254 Factor Bldg., Mailcode 956919, Los Angeles, CA 90095, USA

**Keywords:** squash, Navajo, Diné, lead, cadmium, mining, irrigation water, American Indian, food chain

## Abstract

More than 500 unreclaimed mines and associated waste sites exist on the Navajo Nation reservation as a result of uranium (U) mining from the 1940s through the 1980s. For this study, the impact of U-mine waste on a common, locally grown crop food was examined. The goal of this site-specific study was to determine metal(loid) concentration levels of arsenic (As), cadmium (Cd), cesium (Cs), molybdenum (Mo), lead (Pb), thorium (Th), U, vanadium (V) and selenium (Se) in *Cucurbita pepo* Linnaeus (squash), irrigation water, and soil using inductively coupled plasma-mass spectrometry. The concentrations of metal(loid)s were greatest in roots > leaves > edible fruit (*p* < 0.05), respectively. There were significant differences between metal(loid)s in squash crop plot usage (<5 years versus >30 years) for V (*p* = 0.001), As (*p* < 0.001), U (*p =* 0.002), Cs (*p* = 0.012), Th (*p* = 0.040), Mo (*p* = 0.047), and Cd (*p* = 0.042). Lead and Cd crop irrigation water concentrations exceeded the United States Environmental Protection Agency (USEPA) Maximum Contaminant Levels for drinking water for those metals. Edible squash concentration levels were 0.116 mg/kg of As, 0.248 mg/kg of Pb, 0.020 mg/kg of Cd, and 0.006 mg/kg of U. Calculated human ingestion of edible squash did not exceed Provisional Tolerable Weekly Intake or Tolerable Upper Limit levels from intake based solely on squash consumption. There does not appear to be a food-ingestion risk from metal(loid)s solely from consumption of squash. Safer access and emphasis on consuming regulated water was highlighted. Food intake recommendations were provided. Continued monitoring, surveillance, and further research are recommended.

## 1. Introduction

Metal and metalloid contamination may occur in uranium (U)-mining-impacted areas [1,2,3,4], particularly in agricultural areas [5,6] where metals can migrate into the food chain from anthropogenic sources. The history of U mining on Navajo (Diné) lands began in the 1940s and continued through the 1980s; a considerable legacy of waste remains. A portion of this study examined the locally-raised, subsistence foods that have been reported elsewhere [3,4]. The findings of this current study, further, report data on *Cucurbita pepo* (*C. pepo*), with common names including summer squash, winter squash, and field pumpkin [7,8]. Local studies have documented potential and active contamination of food chain products within U-mining-impacted areas [2,3,4]. The local community has practiced subsistence farming for many generations, which is considered a sacred occupation and is richly steeped in history and stories [9]. Traditional Diné foods, notably squash, their uses, and cultural significance (i.e., squash gourd and various plant organs are used for ceremonial purposes) have been reported in various studies [9,10]. In many American Indian (AI), Alaskan Natives (AN), and world indigenous communities, crops are a vital source of food, possess medicinal uses and properties, and are used as cultural implements for ceremonial purposes. A significant number of AI communities are found to be in close proximity to more than 160,000 abandoned mining areas [11], which have direct detrimental effects on water, food, and other important cultural resources [2,12,13,14,15,16]. 

The *Cucurbita* species ranks among the top-ten crops worldwide; the main producers include China, India, Africa, and North and Central America [17]. Squash is primarily grown for human consumption but has also been used for its seeds (i.e., food source, seed oil). Various world communities also consume its flowers, leaves, and vines. Various cultures value *C. pepo* seeds for traditional medicinal purposes [9,18,19]. In contemporary times, *C. pepo* has been studied for its possible anti-tumor effects [20], as an antihyperglycemic agent [21], and immune-function enhancer [22]. It has been demonstrated to have pharmacological anti-inflammatory, antiviral, and analgesic properties [23]. These traditional and contemporary resources may be threatened by anthropogenic activities that expose crops to dangerous metal(loid)s that negatively impact food sources or have negative health consequences.

U and other metal(loid)s pose significant potential risks for exposure via transfer through the food chain, and their health implications are well documented. Natural U has been shown to co-occur with other metal(loid)s or are a part of its decay series. U is chemotoxic and radiotoxic. It can cause chemical toxicity to kidneys [24] and accumulate in bones [25,26]. Recent studies show an association between U and hypertension, as well as an increased risk for multiple chronic diseases, including diabetes and kidney disease [27]. Metals can have multiple health effects that range from kidney problems from U [28,29] and Cd [30], to neurodevelopmental problems from lead Pb [31], and adverse respiratory effects from V [32]. Arsenic is a metalloid and is a teratogen [33]. Selenium is an essential trace mineral and is also considered a metalloid [34], at high doses are prone to teratogenic effects [35]. Molybdenum [36,37] and Cs [38] are reproductive toxicants and Th has been associated to an increase in cancer risk in high doses [39]. It is important to identify metal(loid)s and other contaminants in food as they may cause deleterious health issues (Pb, Cd, U, and Se) and, in some cases, have long-lasting or permanent effects. Many factors associated with the growth of food, its preparation, and intake can be adjusted to ameliorate these dietary risks. 

Subsistence farming is common on the reservation and has a strong agricultural, historical tradition [40,41,42]. Tribal community members are known to utilize multiple crop plots that have been passed down through many generations [42]. In non-drought conditions, most families rely primarily on natural precipitation for irrigation. Currently, due to a longstanding drought, most families rely more on direct application of irrigation water. Community irrigation water access is a known farming challenge in this region [41,42]. Water sources are primarily unregulated, such as livestock wells, private wells, earthen dams, and rainwater capture. A local study [2] reported that >50% of Diné participants drank from unregulated sources, and more than 80% hauled water despite having public water or regulated water available in their homes. The network of exposures is numerous and complex. For example, the study area also contained mine waste [2], which may contribute to leaching of contaminants into local water sources or may contribute to deposition of windblown metal(loid)s onto soil, plants, or open water sources. There is a risk of exposure to humans consuming livestock which have ingested contaminated crops or wild forage plants. Consumption patterns, such as sharing free food, bartering, or selling local food, also have the potential to impact exposure.

The water- and land-use-pattern-survey data from the current study revealed that people who live near U mining and waste sites may be exposed via agricultural means and was previously suggested by deLemos and associates [2]. The goal of the study was to determine heavy metal (Cd, Cs, Pb, Mo, Th, U, and V) and metalloid (As, Se) concentration levels in squash grown in individual gardens on the Navajo Reservation; soil and irrigation water were also examined. The study objective was to use participant dietary intake information to calculate the estimated ingestion risk exposure to metal(loid)s that have an existing Provisional Tolerable Weekly Intake (PTWI) limit (As, Cd, and Pb) or a Tolerable Upper Limit (UL; Mo, Se, and V). This regional study attempted to address the gaps in knowledge by providing updated, more-detailed crop and irrigation data, provide baseline information on squash harvesters, examine and compare concentrations in *C. pepo* parts, and calculate ingestion exposure risk. The hypothesis is that squash-plant metal exposure primarily occurs via contaminated irrigation water with acknowledgement of the many additional variables that exist (splash from precipitation, wind deposition, duration of crop plot usage).

## 2. Materials and Methods 

Squash crops were sampled and evaluated for As, Cd, Cs, Pb, Mo, Se, Th, U, and V, as were the soil and water within a 3.2-km radius of abandoned U mines and structures. Cohort data was obtained from the Diné Network for Environmental Health (DiNEH) study [2] to identify potential participants whom also participated in current agricultural activities. The remaining participants were selected into the study by word-of-mouth, home visits, and via “chapter” (or community) tribal meetings and events. 

### 2.1. Setting

**Figure 1:**
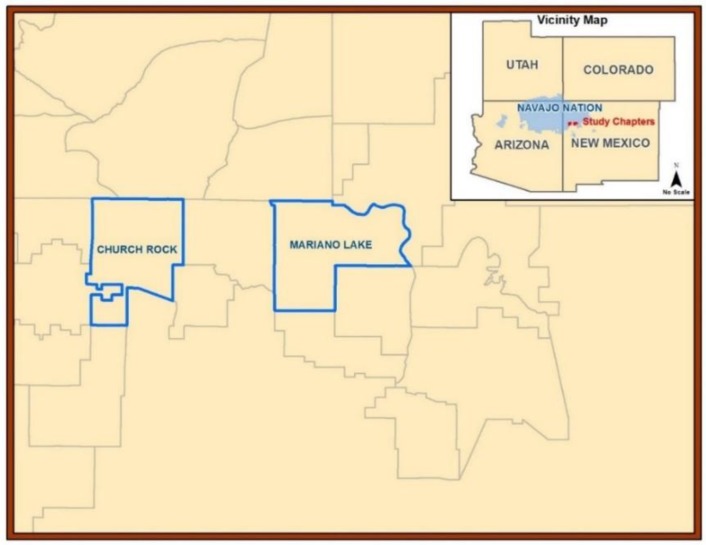
Map of study area in Northern New Mexico: Churchrock and Mariano Lake Chapter Communities of Diné Lands.

The study site is in an arid-to-semi-arid region of the U.S. Southwest on the Navajo reservation. The average elevation is 2192 m. Two community chapters participated and provided agricultural samples. Their combined community land mass was 505 km^2^ (Figure 1). The average precipitation was less than 25 cm per year according to the climate meteorological data in New Mexico reported by the Western Regional Climate Center Western U.S. Climatic Historic Summaries (January 2011–September 2012). In this region the natural occurring mineral deposits consist of U and As [14] and uranyl and vanadate [43]. 

Recruitment activities took place from May through September 2012. Samples were collected during one harvest season from 8 August 2012 to 14 September 2012. The University of California, Los Angeles (UCLA) Institutional Review Board (IRB# 11-001594-CR-00005) and the Navajo Nation Human Research Review Board (#NNR-11.321) approved of this study. This study contributes to a larger parent study, with results reported elsewhere for wild herbal tea [3] and the primary meat staple [4].

### 2.2. Plant and Plant Part Samples

Cultivated, live *C. pepo* consisting of the edible portion, leaves, and roots which were removed from the soil and handpicked with latex-gloved hands, stored in polyethylene (PE) Ziplock^®^ plastic bags, and immediately placed on dry ice. The collection of squash occurred within and around a 1 m radius sample ring from where soil was sampled. The crops were segmented into edible fruit, leaves, and root using sterile, disposable, stainless-steel scalpels. Each crop segment was placed in a PE bag and placed on dry ice. The root was washed non-vigorously using lab-grade (American Society Testing Materials II heavy metal grade) deionized water.

### 2.3. Plant Identification and Nomenclature

Live, parallel plant samples were dried using a plant press in preparation for archive. A log was kept of the samples, plant description, and Global Positioning System (GPS) location. Color photographs were taken of the samples. Plants were identified and archived at the University of New Mexico (UNM) Herbarium. 

### 2.4. Human Harvester Questionnaire Data

Two questionnaires were utilized to gather harvester information. A general questionnaire, the Diné Plant–Animal–Human Questionnaire (DPAHQ), was used to collect harvester demographic information and basic overall food use. The Diné Crop Intake Questionnaire (DCIQ) collected more detailed crop use information, such as duration of plot use, type and amount of water irrigation used, frequency and amount of squash consumption, details of crop storage, and incidence of crop sharing and sale.

### 2.5. Soil Samples

To avoid cross contamination, a coated-core sampler (Art’s Manufacturing and Supply, Inc. (AMS) Core Sampler^®^, American Falls, ID, USA) was used. A stainless-steel hand auger was used to collect soil samples. The core was lined with a PE liner (AMS Core Sampling Mini-kit^®^, American Falls, ID, USA). Samples were taken from the topsoil (0–15 cm) and subsoil (15–91 cm) using a random, zig-zag pattern. Two depths were obtained of agricultural soils to encompass the plough zones. One hundred grams each of topsoil and subsoil were collected. Various physiochemical properties, such as temperature, pH, Munsell color, moisture, and depth, were obtained. Each soil sample was paired with squash vegetable and irrigation water samples. Metal(loid) concentrations (As, Cd, Cs, Pb, Mo, Se, Th, U and V) were determined in top and subsoils.

### 2.6. Water Samples

Water samples, obtained from a faucet or spigot when available, were collected as first-draw samples. Standing water samples from multiple water vessels were collected as composite water grab samples. Two hundred and fifty milliliters (mL) of water were collected in lab-grade, HM-analysis, PE water bottles. Water temperature and pH data were collected. Nitric acid (HNO_3_) preservative was added to each sample and immediately placed on dry ice for transport. A duplicate was obtained for each sample and a blank for each sample session. Water samples were determined for metal(loid)s (As, Cd, Cs, Pb, Mo, Se, Th, U, and V).

### 2.7. Global Positioning System Data

To interpret and provide location information for the samples, Global Positioning System (GPS) instrumentation (Trimble Navigation Limited, Westminster, CO, USA) was utilized. Field samples were marked and geocoded using a 2008 Trimble R GeoXT (Trimble Navigation Limited, Westminster, CO, USA) [44]. Differential correction within 72 hours of data capture was completed using GPS Pathfinder Office version 5.30 (Trimble Navigation Limited, Westminster, CO, USA). Spatial proximity analysis between the samples and mine sites and documentation of sample locations were completed via Geographic Information Systems (GIS) analysis.

### 2.8. Sample Analysis

The crop, soil, and irrigation water samples were prepared and analyzed by the UNM Analytical Chemistry Laboratory, Earth and Planetary Sciences Department utilizing ICP-MS (PerkinElmer NexION 300D, Waltham, MA, USA). Sample analysis has been documented and reported in whole in previous publications [3,4]. Aqueous samples were prepared by acid digestion protocol in which 50 mL sample were transferred into digestion tubes. Five ml ultra-high purity (UHP) nitric acid (HNO_3_) and 2 mL hydrogen peroxide (H_2_O_2_) were added. Samples were heated gradually up to 95 °C. After digestion was completed, water samples were transferred into 50 mL volume metric flasks and brought to volume using 18 mega ohm water. Solid samples (soils and plants) were prepared by weighing 2 grams into digestion tube. Five mL UHP HNO_3_ and 2 mL H_2_O_2_ were added and samples were heated gradually up to 95 °C. After digestion was completed, water samples were transferred into 50 mL volume metric flasks and brought to volume using 18 mega ohm water. With each batch of samples, a reagent blank (3 mL HNO_3_) was digested and upon completion, the reagent blank sample was transferred into 50 mL volume metric flasks and brought to volume using 18 mega ohm water. With all digestion protocols, samples were filtered using a 0.45-micron filter before transferring into 50 mL volumetric flasks.

The method detection limits are U: 0.008 μg/L, Pb: 0.008 μg/L, Th: 0.012 μg/L, Mo: 0.02 μg/L, Cd: 0.1 μg/L, V: 0.1 μg/L, As: 0.3 μg/L, and Se: 1.3 μg/L. Three replicates of each sample were measured. Only a subset of metals were tested. Certified reference materials were analyzed as quality control measures utilizing SRM 1573a (tomato leaves; NIST, Gaithersburg, MD, USA) and SRM 2709a (San Joaquin soil; NIST, Gaithersburg, MD, USA) yielding the following values: Cd: 1.47 ± 0.11 (certified tomato leaves value: 1.52 ± 0.04), V: 0.94 ± 0.07 (certified tomato leaves value: 0.84 ± 0.01), Cd: 0.64 ± 0.09 (certified soil value: 0.37 ± 0.02), V: 83.20 ± 7.70 (certified soil value: 110 ± 11). The precision of the results demonstrated relative standard deviations varying from 7.1% to 13.8%.

### 2.9. Statistical Analysis

The statistical analysis software used was provided by the IBM SPSS for Windows version 23 (IBM, Armonk, NY, USA) [45]. Heavy metal and metalloid concentrations in plant and soil samples were reported in milligrams per kilogram (mg/kg). Metal(loid) water concentrations were reported in micrograms per liter (μg/L). To summarize data, percentages, ranges, means, standard deviations, and medians were reported. To compare the differences between heavy metal levels in squash parts, soil, and water, *t* tests were used. A *p* value of <0.05 was considered significant.

## 3. Results

### 3.1. Human Harvester Questionnaire Data

The mean age of harvesters was 61.50 ± 23.34 years, and they had lived in the current harvesting location for a mean of 51.50 ± 9.19 years. Gender groups were equally divided between men and women. Squash was consumed by harvesters for a mean of 17.50 ± 19.09 years. On average, *C. pepo* was eaten 1.27 times per week for 5.5 months per year. Participants used the crops primarily for human consumption (with and without the rind in young squash and without the rind in mature squash) along with, to a lesser extent, livestock feed and several cultural uses. Crop plots have reportedly been in use for multiple generations (56.0 ± 35.4 years). Current harvesters reported yearly plot use without fallow. Harvesters used a combination of hand and mechanical techniques or relied exclusively on agricultural machinery for planting. All participants reported using non-commercial, or heirloom, seeds. No participant used commercial soil amendments or fertilizers. One harvester reported using squash leaves for horse feed (other crop parts were reserved for sheep); successively, the horse manure was used as a natural fertilizer for squash. In all cases, the harvesters reported storing squash in their home during and after harvesting seasons. The squash crop was shared cost-free by all participants with nearby community members, across and off the reservation. One-half of participants reported selling the squash within their community, across the reservation, and at off-reservation sites. On average, two water sources (consisting of any combination of public water, undetermined water source, captured rainwater, and natural precipitation) were utilized by each participant. The mean crop plot size was 0.42 ± 0.29 hectares. While participants did not report currently drinking irrigation water, one participant did report consuming irrigation water in years past. In most instances, irrigation water was stored in plastic or metal vessels. Water was transported to the crop areas from multiple sources, such as private wells; from the home, public water system; from rainwater collection sites; or via natural precipitation. 

### 3.2. Metal(loid)s in Irrigation Water 

Of all mean water concentration levels, Cd (44.73 ± 3.77 μg/L) and Pb (16.2 ± 2.68 μg/L) exceeded the USEPA Maximum Contaminant Levels (MCLs; Table 1) by 895% (Cd) and 108% (Pb). As and Se were below the MCLs [46] including U (7 μg/L). Water concentrations of Th were negligible. Of the metal(loid)s that have a drinking water standard, U, As, and Se were each less than 50% (range 23.4–43.6%) of the MCLs. The remaining metals are not covered under the established MCLs (Cs, Mo, Th and V).

### 3.3. Metal(loid)s in Soil and Crop Plant Tissue

In most instances, the mean soil metal(loid) concentrations had higher values compared to edible *C. pepo* (Table 2). V concentrations in soil (21.27 ± 11.22 mg/kg) were greater than U, Cs, As, Th, Se, Pb, or Mo (range 1.01–8.42 mg/kg), and the Cd concentration was less than 1 mg/kg (0.53 ± 0.29). The mean soil pH was 6.3 ± 0.4. Metal(loid) concentrations were greater at deeper soil depths but failed to show statistical significance (*p* > 0.05).

For five of the co-metal(loid)s (As, Cs, Pb, U, and V), the mean concentrations were greater in roots than in the leaves or fruit. For Cd and Th, the mean concentrations found in the leaves were greater than the root or fruit. The concentrations of Mo and Se were greater in the leaves than the fruit or root. There were statistically significant increased concentrations in the leaves compared to the edible squash for the majority of the metal(loid)s: V, Th, U, Se, Cd (*p* < 0.001), Pb, and As (*p* < 0.05). Overall, U concentration levels were lowest of all edible fruit (0.006 mg/kg) and soils (1.01 mg/kg) determined.

### 3.4. Comparison of Metal(loid)s by Crop-Plot-Production Years

The mean squash-crop-plot-production years stratified into two categories: Less than 5 years and greater than 30 years of active plot usage (overall mean: 19.4 ± 13.9). Table 3 shows significant differences were found between samples from the lower and higher plot-year categories with higher soil metal(loid) concentration levels for greater plot usage production years for V (*p* = 0.001), As (*p* < 0.001), U (*p* = 0.002), Cs (*p* = 0.012), Th (*p* = 0.040), Mo (*p* = 0.047), and Cd (*p* = 0.042).

### 3.5. PTWI Human Intake Calculations

The human intake calculations were completed for As, Cd, and Pb; the PTWI [48,49] were calculated as:

PTWI = daily intake of metals = ∑[concentration of metals in edible squash × mean of squash intake (grams per person per day)], weekly intake of metals = daily intake x seven days/week, weekly intake per body weight (kg) (PTWIs) = weekly intake/reference body weight (60 kg)(1)

### 3.6. Human Intake Calculations for As, Cd, and Pb

The weekly intake calculation for *C. pepo* was 0.87 μg/kg of Cd, 5 μg/kg of As, and 11 μg/kg of Pb (Table 4). The percentages below the PTWI for edible squash were 34% for As, 12.4% for Cd, and 44% for Pb. PTWIs do not currently exist for Cs, Mo, Se, Th, U, and V. 

### 3.7. Human Intake Calculations for Mo, Se, and V

The daily intake calculations for V were 3 μg, 9.3 μg of Mo, and 19.3 μg of Se (Table 5). There exist no Reference Dietary Intake (RDI) or recommended dietary allowance (RDA) for V [50]. However, the tolerable UL for V was 0.2%. The results demonstrated that 21% of the RDA for Mo [50] and 35% of the RDI for Se were consumed [51]. The calculated daily intake for Mo and Se were both below the UL (0.46% and 4.8%). RDIs, RDAs, and ULs do not exist for As, Cd, Cs, Pb, Th, and U.

## 4. Discussion

The mean metal(loid) concentration levels were generally higher in plant roots than plant leaves and edible squash. With the exception of Pb, soil concentrations were greater than edible squash, leaves, and squash roots. These findings were consistent with similar agricultural studies [52,53,54,55,56]. One regional squash study demonstrated that HM leaf concentrations were the greatest of all crop parts [57]. In other plant species, U had a tendency to accumulate in the roots of plants [58]. Species of the *Brassica* family, leafy vegetables, and root crops had greater concentrations of U than grass species. This is in agreement with other studies demonstrating that metal-dependent uptake is determined by plant species [57] and, even, plant part [52,55]. Leafy vegetables contained higher Pb concentrations than other non-leafy vegetables, which is hypothesized to be primarily resulting from air deposition [52,56].

The MCLs [46] were exceeded for Cd (44.7 ± 3.8) and Pb (16.2 ± 2.7) in squash irrigation water. The metals that have a water standard (As, Se, and U) did not exceed water MCLs [46,47]. Despite the elevated Pb and Cd concentration levels found in irrigation water, the edible portions of *C. pepo* contained lower corresponding concentration levels (range: 0.02–0.25 mg/kg). In most cases, it appears that the metal(loid)s primarily translocate from the soil to the roots or leaves. According to the harvester questionnaire water data, the metal concentration levels for one participant that utilized repurposed metal irrigation vessels contained higher Cs, Pb, Cd, and Mo concentrations in water than those that did not use repurposed metal tanks. The repurposed metal tanks were obtained from the Fort Wingate Army depot, a known contaminated munitions site [59]. At the time of sample collection, all study participants were informed of the importance of exclusively using food-grade water vessels for crop irrigation and were discouraged from consuming water from metal, non-food-grade water containers or those vessels of unknown origin or unpronounceable labeling, information comparable to recommendations of the NNEPA [60]. Agricultural water access remains a major issue [42], and residents may be pressed to use available water sources, which are often unregulated. Even though participants did not report currently drinking the irrigation water, it is still a cause for concern as it may be used for drinking, bathing, cooking, and water for locally consumed livestock. In the same study communities, deLemos [2] reported that 100% of study participants hauled (transported) water despite having a home, public water source. Up to 30% of Diné homes lack access to regulated water. The lack of water infrastructure, history of extensive U mining, and co-occurrence of other metal(loid)s with U continue to be perceived as major health issues that need to be addressed on the Navajo reservation [14]. 

In all soil, edible crop, and water samples, U concentrations levels were low and the lowest in each category. However, in U mining impacted areas, the importance of determining other co-metal(loids) and contaminants is as important as focusing on the primary metal mined. With the current study, associated metals and contaminants were found to be elevated and may be contributing to health risk in food and water. In a regional study, Hoover et al. [14] emphasized the risk of co-exposures to multiple contaminants in U impacted areas. Determining the interactions between contaminants and the cumulative adverse health risks of multiple contaminants are needed. More research is needed to determine and evaluate co-contaminant exposures, allowing improved characterization of health impacts. 

According to participant questionnaire data, the mean number of years of squash consumption was 17.50 ± 19.09 years. The amount of time residing within the harvest area was 51.50 ± 9.19 years, and the crop-plot-production years were extensive (56.0 ± 35.4). The number of production years of a crop plot may also exhibit a relationship. This study demonstrated that increased years of squash production history correlated with greater metal(loid) concentrations for seven of the contaminants examined (As, Cs, Cd, Mo, Th, U, and V). Huang and Jin [61] also found that there were greater soil HM concentrations with increasing field production years (5–10 versus 11–20 years); however, the crop producers reported using chemical fertilizers. The current study was dissimilar in that study participants reported no use of chemical fertilizers. The length of exposure related to participating in agricultural activities was extensive among this cohort. Exposure to metal(loid)s can occur during various stages of agricultural activities, including planting, cultivating, weeding, harvesting, food preparation, cooking, and storing crops. One study reported that food processing or cooking (via boiling, grilling, or baking) may alter Se levels, though research has yet to determine in which direction [62]. Another study found that As dissociates in water at a range of temperatures (via boiling) [63]. All study harvesters reported storing squash in their homes upon harvest, for at least 5.5 months out of the year before it was consumed. Determining the level and extent of exposure at various stages of harvesting and storage along with the impact of food preparation and cooking (i.e., consumption of squash with and without the rind) is an area of future investigation.

### 4.1. Human Harvester Data and Implications

In the Diné community, squash is a supplemental, household, subsistence food crop. It is a common food-crop staple. The typical Diné serving size of squash (300 g) was determined based on a comprehensive traditional food study by Wolfe et al. [10]. The typical squash intake reported by the harvesters enrolled in this study was 1.27 times per week. No dietary intake recommendations exist for some of the studied heavy metals (U, Th, and Cs), but PTWIs exist for As (15 μg/kg), Cd (7 μg/kg), and Pb (25 μg/kg; see Table 4). The PTWI is established by the United Nations Food and Agriculture Organization in conjunction with the World Health Organization and is known as the Joint Expert Committee on Food Additives [48,49]. PTWI is defined as “a chemical with no intended function as an estimate of the amount of the chemical that can be ingested weekly over a lifetime without appreciable health risk” [64] (p. 2588).

The RDI for Se is 55 μg/day, and the tolerable UL is 400 μg/day (see Table 5; Food and Nutrition Board (FNB)) [51]. The RDI and RDA are defined as the dietary intake levels sufficient to meet the nutrient requirements of 97–98% of healthy persons of a particular gender and life stage [65,66]. The UL is the “highest level of nutrient intake that is likely to pose no risk of adverse health effects for almost all individuals in the general population” [65] (p. 364). The RDA for Mo is 45 μg/day, and the UL is 2000 μg/day [50]. For V, there is no RDI or RDA; however, the UL is 1800 μg/day [50]. The UL was not exceeded for any of the metal(loid)s, thus our calculations support that squash consumption alone does not pose a significant risk of exposure to Mo, Se, or V. This study was comprised exclusively of adults; therefore, the calculations are based only on adult food intake.

The PTWIs for As, Cd, and Pb and the ULs for Mo, Se, and V were not exceeded solely from oral consumption of squash. The percentage of PTWI for each metal(loid) (As, Cd, and Pb) was less than 45%, and the percentage of UL for each metal (Mo and V) and metalloid (Se) was less than 5%. The RDAs and RDIs for Se and Mo may have been met by other foods consumed in the diet, but this study only focused on one subsistence crop of the whole diet. However, as the Se RDI and Mo RDA were not met, the researchers suggest increasing intake of foods higher in Se and Mo to meet the recommended requirements, such as meat, grains (Se) [67], legumes, and nuts (Mo) [50]; consultation with a health care provider or dietician is advised. Various animal products, vegetables, and fruits contain low Mo [50], and the amount of Se in the soil is reflective of that found in harvested food [62]. Meats tend to have more Se than plants [68] and reflects the animal consumption by foraging [62]. In a portion of the current cohort, a meat staple study found that Se RDI and Mo RDA were exceeded (though ULs were not exceeded) due to sheep organ meat consumption (particularly liver) [4]; the investigators do not recommend consuming more than the UL. Therefore, collective food intake should be considered in all cases and assessed. The researchers recommend determining metal concentrations in other popular crops (corn [*Zea mays*] and beans [*Phaseolus vulgaris*]) grown in this mining impacted region. This study could not include corn and bean analysis due to low sampling numbers. Diversification of diet has been suggested [4] to mitigate the consumption of food containing metal(loid)s in impacted areas. Unregulated irrigation water is still a major concern and should be avoided for use in food preparation and cooking. Food-grade water vessels should be exclusively used for any purpose associated with the food chain. Whether there is an increased exposure risk from bathing and cooking is a concern that requires further research. It would be ideal to study exposure risk attributed to all the various types of foods (subsistence, non-subsistence, etc.) and water consumed collectively, in order to further establish the health impacts of co-occurring contaminants. Tailored research is also suggested to examine the exposure of metal(loid)s to specific populations, such as the very young, those of advanced age, those that are contemplating or are pregnant, lactating women, or those with chronic health conditions (i.e., renal or liver disease or failure, diabetes, altered immune function or autoimmune disorders). Currently there exist no food guidelines that address various metal exposures such as Cs, Th, and U.

### 4.2. Limitations

Study irrigation water may have been transported or collected from multiple sources. These water sources may be more or less contaminated than the home sampling areas. In addition, water metal(loid) concentrations change over time, thus our samples provide concentration levels at only one point in time. Future research should strive to incorporate longitudinal examinations. Reevaluation in series or over multiple seasons would be beneficial and provide an improved understanding of the variations attributed to seasonal (e.g., windy seasons) or climatic factors (i.e., drought). The collected *C. pepo* samples demonstrate the metal(loid) concentration levels indicative of soil adhering to plants and reflect the natural state in which they were found. Chambers and Sidle [69] found comparable metal concentration levels with unwashed and washed plant samples. Air monitoring studies would provide insight into the extent and amount of metal(loid)s adhering to plants via aerosolization. Lastly, the large number of statistical tests performed forces a conservative interpretation.

### 4.3. Recommendations

The need for further study and continued monitoring and surveillance is important and should not be undervalued. The investigators encourage and emphasize: the continued use of water and soil maps recommended by deLemos et al. [2] in high risk areas, water education information provided by the NNEPA [60], and food study recommendations [3,4]. For the future, the researchers recommend the use of human biological samples to link food exposures to food contaminants. The involvement of the Navajo tribe in all aspects of the study is highly encouraged and beneficial. The MCLs for Cd and Pb were found to be grossly exceeded. Thus, ingestion of unregulated water remains a major concern. The field observations in this study area highlight the continual recommendations to avoid using non-food-grade water vessels for any aspect of the food chain. Furthermore, measures to improve access to clean, safe water are recommended [14]. This study found that squash consumption alone is a low food risk for As, Cd, Pb, Se, Mo, and V. The researchers recommend continued research and monitoring in this high U-impact area. A discussion of Cs, Th, and U tolerances in the dietary intake are beyond the scope of this paper. However, food intake guidelines are needed for these metals to address the food and water contaminants as they may pose adverse health risks in mining impacted areas. Further, risk assessment tools are needed that will evaluate cumulative risk of health impacts of multiple co-contaminants. Other recommendations include involving communities, the leadership, and health policy makers in addressing safe accessibility to food, water, and cultural resources. 

## 5. Conclusions

The study showed that various elements can accumulate differently in various parts of the squash plant. The concentrations of metal(loid)s were found in greatest concentrations in roots > leaves > edible fruit. In most instances U concentration levels were predominately low in most sample categories, nevertheless elevated concentrations of metals in soil and water (Cd and Pb) were demonstrated to be the main metal contaminants in squash. There was a positive relationship between the greater number of years of crop production (>30 vs. <5) for most of the metal(loid)s examined. However, there does not appear to be a dietary food risk of metal(loid) ingestion based solely on the consumption of squash.

## Figures and Tables

**Table 1:** Metal(loid) concentrations in *Cucurbita pepo* irrigation water.

Metal(loid)	Irrigation Water (Mean ± Standard Deviation, μg/L, *n* = 4)	Maximum Contaminant Levels (MCLs)	% Above or Below MCLs
As	3.80 ± 0.69	10 ^a^	38
Cd	44.73 ± 3.77	5 ^a^	895
Cs	0.46 ± 0.10	^b^	^b^
Pb	16.18 ± 2.68	15 ^a^	108
Mo	1358.07 ± 125.79	^b^	^b^
Se	21.81 ± 11.34	50 ^a^	43.6
Th	ng	^b^	^b^
U	7.03 ± 6.12	30 ^a,c^	23.4
V	26.44 ± 0.61	^b^	^b^

Note: ng: negligible; ^a^ USEPA (2009) Maximum Contaminant Levels (MCLs); ^b^ No established USEPA drinking water standard; ^c^ Navajo Nation Environmental Protection Agency (NNEPA) Surface Water Quality Standards [47].

**Table 2:** Metal(loid) concentrations in *C. pepo* plant parts and soil.

Metal(loid)	Squash Fruit (Mean ± Standard Deviation, mg/kg, *n* = 12 *)	Squash Root (Mean ± Standard Deviation, mg/kg, *n* = 4 *)	Squash Leaves (Mean ± Standard Deviation, mg/kg, *n* = 6 *)	Soil (Mean ± Standard Deviation, mg/kg, *n* = 14 *)
As	0.116 ± 0.086	0.327 ± 0.0000 ^c^	0.243 ± 0.014	1.96 ± 0.66
Cd	0.020 ± 0.008 ^b^	0.029 ± 0.000 ^c^	0.100 ± 0.036	0.53 ± 0.29
Cs	0.069 ± 0.039	0.136 ± 0.129	0.112 ± 0.091	1.78 ± 1.07
Pb	0.248 ± 0.092	8.90 ± 17.13	0.450 ± 0.165	6.89 ± 0.85
Mo	0.170 ± 0.041 ^a^	0.132 ± 0.0134 ^c^	0.203 ± 0.056	8.42 ± 5.69
Se	0.354 ± 0.133 ^b^	0.160 ± 0.0898 ^c^	1.26 ± 0.52	5.98 ± 2.62 ^d^
Th	0.049 ± 0.042 ^a^	0.121 ± 0.062	0.246 ± 0.110	3.84 ± 0.63
U	0.006 ± 0.006	0.035 ± 0.021	0.023 ± 0.010	1.01 ± 0.58
V	0.054 ± 0.021	1.14 ± 0.79	0.607 ± 0.325	21.27 ± 11.22

Note: * The number of samples vary: ^a^ *n* = 11; ^b^ *n* = 10; ^c^ *n* = 2; ^d^ *n* = 6.

**Table 3:** Differences in metal(loid) concentrations based on plot-production years.

Metal(loid)	Plot Production < 5 Years (mg/kg, *n* = 6)	Plot Production > 30 Years (mg/kg, *n* = 8)	*p*
As	1.33 ± 0.10	2.43 ± 0.44	<0.001
Cd	0.32 ± 0.33	0.69 ± 0.12	<0.05
Cs	1.01 ± 0.20	2.36 ± 1.10	<0.05
Pb	6.98 ± 0.61	6.82 ± 1.04	>0.05
Mo	4.40 ± 6.59	11.44 ± 2.19	<0.05
Se	5.98 ± 2.62	ng	>0.05
Th	3.45 ± 0.29	4.14 ± 0.67	<0.05
U	0.52 ± 0.07	1.38 ± 0.51	<0.01
V	11.59 ± 2.14	28.53 ± 9.49	<0.001

Note: ng = negligible.

**Table 4:** Calculated dietary exposure to As, Cd, and Pb from *C. pepo* consumption.

Metal(loid) *	Weekly Intake (μg/kg of BW ^a^)	PTWI (μg/kg of BW ^a^)	% Below the PTWI
As	5.14	15	34.3
Cd	0.87	7	12.4
Pb	11.06	25	44.2

Note: * There are no PTWIs for Cs, Mo, Se, Th, U and V. ^a^ Body weight (reference weight 60 kg).

**Table 5:** Calculated dietary exposure to Mo, Se, and V from *C. pepo* consumption.

Metal(loid) *	Daily Intake (μg)	RDI, RDA or UL (μg/Day)	% Below the RDI, RDA or UL
Mo	9.27	RDA: 45 UL: 2000	RDA: 20.6 UL: 0.46
Se	19.27	RDI: 55 UL: 400	RDI: 35.04 UL: 4.8
V	2.93	UL: 1800	UL: 0.16

Note: * There are no RDI, RDA, or ULs for As, Cd, Cs, Pb, Th and U.
